# Cuproptosis regulatory genes greatly contribute to clinical assessments of hepatocellular carcinoma

**DOI:** 10.1186/s12885-022-10461-2

**Published:** 2023-01-07

**Authors:** Changwei Ke, Shejiao Dai, Fangshi Xu, Jia Yuan, Shuting Fan, Yang Chen, Longbao Yang, Yong Li

**Affiliations:** 1 Department of Emergency, The Second Affiliated Hospital of Xi’an Jiaotong University, Xi’an, Shaanxi Province China; 2 Department of Gastroenterology, The Second Affiliated Hospital of Xi’an Jiaotong University, No. 157, West Five Road, Xi’an, 710004 Shaanxi China; 3 Department of Medicine, Xi’an Jiaotong University, Xi’an, Shaanxi Province China; 4 Department of Outpatient, The Second Affiliated Hospital of Xi’an Jiaotong University, Xi’an, Shaanxi Province China

**Keywords:** Cuproptosis, Hepatocellular carcinoma, Risk signature, Prognosis, DLAT

## Abstract

**Background:**

Hepatocellular carcinoma (HCC) is a common abdominal cancer with dissatisfactory therapeutic effects. The discovery of cuproptosis lights on new approach for cancer treatment and assessment. So far, there is extremely limited research investigating the roles of cuproptosis-related (CR) genes in cancers.

**Methods:**

A novel CR risk signature was constructed using the Lasso regression analysis. Its prognostic value was assessed via a series of survival analyses and validated in three GEO cohorts. The effects of CR risk signature on tumor immune microenvironment (TIM) were explored through CIBERSORT, ESTIMATE, and ssGSEA algorithms. Using GESA, we investigated its impacts on various metabolism process. The somatic mutation features of CR signature genes were also explored via cBioPortal database. Using tumor mutation burden, expressions of immune checkpoints, TIDE score, IMvigor 210 cohort, and GSE109211 dataset, we explored the potential associations of CR risk score with the efficacy of immune checkpoint inhibitors (ICIs) and sorafenib. Finally, the biofunctions of DLAT in HCC cells were ascertained through qPCR, immunohistochemistry, colony formation, and Transwell assays.

**Results:**

FDX1, DLAT, CDKN2A and GLS constituted the CR risk signature. CR risk signature possessed high prognostic value and was also applicable to three validation cohorts. Meanwhile, it could improve the accuracy and clinical making-decision benefit of traditional prognostic model. Moreover, high CR risk was indicative of unfavorable anti-tumor immune response and active metabolisms of glycolysis and nucleotide. As for therapeutic correlation, CR risk score was a potential biomarker for predicting the efficacy of ICIs and sorafenib. Through qPCR and immunohistochemistry detection in clinical samples, we reconfirmed DLAT was significantly upregulated in HCC samples. Overexpression of DLAT could promote the proliferation, migration, and invasion of HepG2 and HuH-7 cells.

**Conclusions:**

The novel CR risk signature greatly contributed to the clinical assessment of HCC. Cuproptosis regulatory gene DLAT possessed cancer-promoting capacities and was expected to be a promising therapeutic target for HCC.

**Supplementary Information:**

The online version contains supplementary material available at 10.1186/s12885-022-10461-2.

## Background

Hepatocellular carcinoma (HCC), a common abdominal tumor typically originating from the cirrhotic liver, is the fourth most common cause of cancer-related death worldwide, leading to over 400,000 deaths throughout China in 2021 [1, 2]. Although unremitting efforts have been devoted to the diagnosis and treatment of HCC, patients still suffer from poor prognoses. In China, more than half of the patients are diagnosed with advanced disease at their first visit, and their 5-year overall survival rate (OSR) is < 12.5% [3]. Surgical excision offers the only possibility for a cure [4]. However, only 34 to 70% of patients may be suitable for hepatic resection; this limitations cause the overall postoperative mortality to reach up to 3% [5]. Moreover, molecular target therapy (MTT) and immune checkpoint inhibitors (ICIs) exhibit limiting improvements in overall survival (OS). For instance, the median OS of sorafenib an approved first-line agent for advanced HCC, is merely 14.7 months [6]. Additionally, only a minority of patients receiving ICIs achieve the treatment response. Nivolumab and pembrolizumab commonly produce a 15–20% rate of objective remissions [7]. These observations underscore the urgency and importance of widening therapeutic strategies and refining clinical assessments. Recently, cuproptosis, a novel form of programmed cell death (PCD), has been a topic of interest in HCC treatment.

Programmed cell death exceedingly expands the anti-cancer arsenal. With the discovery of ferroptosis, necroptosis and pyroptosis, we have obtained a deeper understanding of carcinogenic mechanism and clinical assessment of multiple cancers [8–10]. For example, SLC7A11 the catalytic subunit of Xc- system in ferroptosis could promote malignant biological properties of renal carcinoma cells [9]. Ferroptosis regulator SLC1A5 exhibits its cancer-promoting abilities by activating the mTORC1 signaling pathway [10]. In 2022, Tsvetkov, P et al. has reported noteworthy research on copper-mediated cell death, namely ‘Cuproptosis’ [11]. Mechanistically, metal reductase FDX1 is activated owing to the accumulation of intracellular copper ions. Subsequently, FDX1 mediates the lipoylation of the tricarboxylic acid cycle (TCA) proteins, thereby inducing the oligomerization of lipoylated proteins with the aid of copper ions. Considering that the immense potentials of cuproptosis in cancer treatment, the use of Cu ionophores has been proposed to be an emerging technological approach for targeting cancer cells [12].

The discovery of cuproptosis has attracted considerable interest across the oncology community. Several scholars have commented on this remarkble finding and regarded it as a new bellwether for cancer treatment [13–15]. Nevertheless, limited studies have probed into the roles of cuproptosis regulatory genes in cancers, which is the original aim of this research. In the present study, we sought to construct a novel risk signature based on 17 core CR genes using Lasso regression analysis. Moreover, we intended to investigate its great prognostic value and the abilities for indicating the immune microenvironment, metabolic reprogramming and therapeutic outcomes. Our findings provided novel and valuable evidence of the therapeutic potential of utilizing cuproptosis for treating HCC.

## Materials and methods

### Data source

We obtained the gene expression data and clinical information from TCGA, ICGC and GEO public databases. Owing to the inadequate number of normal samples in TCGA-LIHC cohort (*n* = 50), we added 110 normal liver tissue samples from GTEx database (https://xenabrowser.net/datapages/) to equilibrize the sample sizes of tumor and normal tissues. All transcriptome data was standardized by log2 (FPKM + 1) transformation. The clinical characteristics of TCGA, ICGC and GEO cohorts were presented in Supplementary Table 1–2.

### Cuproptosis-related gene set

Reportedly, protein lipoylation in TCA cycle triggers the onset of cuproptosis via FDX1 mediation. Accordingly, based on the findings of the study by Tsvetkov, P et al. [11], we selected 17 critical cuproptosis regulatory genes for further analysis. The CR genes and their functions in the cuproptosis process were shown in Table 1. We constructed the protein–protein interaction (PPI) network of CR genes using the STRING database (https://string-db.org/) [16] and Cytoscape (version 3.71) software [17]. The biological function analyses of 17 cuproptosis regulators were performed via the DAVID database (https://david.ncifcrf.gov/) [18].

**Table 1:** Seventeen critical genes involved in cuproptosis process

Gene symbol	Description
FDX1	Ferredoxin 1
LIPT1	Lipoyltransferase 1
LIAS	Lipoic acid synthetase
DLD	Dihydrolipoamide dehydrogenase
PDP1	Pyruvate dehydrogenase phosphatase catalytic subunit 1
DLAT	Dihydrolipoamide S-acetyltransferase
PDHA1	Pyruvate dehydrogenase E1 subunit alpha 1
PDHB	Pyruvate dehydrogenase E1 subunit beta
DBT	Dihydrolipoamide branched chain transacylase E2
GCSH	Glycine cleavage system protein H
DLST	Dihydrolipoamide S-succinyltransferase
SLC31A1	Solute carrier family 31 member 1
ATP7A	ATPase copper transporting alpha
ATP7B	ATPase copper transporting beta
MTF1	Metal regulatory transcription factor 1
CDKN2A	cyclin dependent kinase inhibitor 2A
GLS	Glutaminase

### Consensus clustering analysis

We applied consensus clustering for identifying the distinct prognostic patterns based on the features of CR expressions. This procedure was performed using the ‘ConsensusClusterPlus’ package in R software (version 4.1.2) and was based on the algorithm of cumulative distribution function (CDF).

### Establishment of CR risk signature

WE constructed a novel CR risk signature through two steps. First, CR differentially expressed genes (DEGs) were screened out using the ‘Limma’ package in R software (version 4.2.0). The screening criteria were as follows: adjusted *p*-value < 0.05 and absolute value of log_2_FC ≥ 0.58 (1.5 fold difference in gene expression). Second, we used CR DEGs to accomplish the modeling process through the Lasso regression analysis using the ‘glmnet’ R package. This process was performed using the sevenfold cross-validation scheme.

### Prognostic analysis

The optimal cutoff value of the CR risk score was determined using the Cutoff Finder online tool (http://molpath.charite.de/cutoff) [19]. The prognostic differences between high- and low-risk groups were compared based on the Kaplan–Meier method. Independent prognostic factors of HCC were identified using the Cox univariate and multivariate analyses. The accuracy of predicting OSR was assessed using the receiver operating characteristic curve (ROC). Decision curve analysis (DCA) was utilized for determining whether CR risk score could elevate clinical-decision benefit of traditional prognostic models. Furthermore, we conducted clinical subgroup analyses to evaluate the prognostic stratification ability of the CR model in HCC patients with different disease stages. Owing to the low number of samples in M1 (*n* = 3) and N1 stages (*n* = 3), clinical subgroup analyses were not applied to these subgroups. Using multiple logistic regression, a nomogram comprising of clinical stage and CR risk level were constructed to predict the OSR of individual at 1,3, and 5 years. The calibration curve was used to test its predictive accuracy.

We selected the GSE14520, GSE116174 and ICGC-LIRI cohorts to validate the prognostic value of CR risk signature. Survival difference analysis and ROC were both conducted in each validation cohorts.

### Immune and mutational analysis

The CIBERSORT algorithm was performed to quantize the infiltration levels of 21 immune cells in each HCC sample [20]. As described in previous studies [21, 22], ssGSEA (single-sample gene set enrichment analysis) method was employed to calculate the activities of ten immune-related signaling pathways. The ESTIMATE method is an effective approach for inferring the fraction of stromal and immune cells in tumour samples using gene expression [23]. By this method, immune score, stromal score and tumor purity of each HCC sample can be calculated. The cBioPortal database (http://cbioportal.org) [24] provided the somatic mutational frequency and patterns of CR signature genes across four HCC projects (*n* = 973 samples).

### GSEA

GSEA (Gene Set Enrichment Analysis) was used to investigate the influence of CR risk score on multiple metabolic processes, including glycolysis, nucleotide, cholesterol, glutamine, and fatty acid metabolisms. The MSigDB database (https://www.gsea-msigdb.org/) provided the used gene sets. The detailed descriptions of metabolic gene sets were presented in Supplementary Table 3. The phenotype labels were set as high-CR risk versus low-CR risk samples. The number of permutations was set at 1000, and there was no collapse in gene symbols.

### Therapeutic correlation analysis

WE explored the potential associations of CR risk score with the efficacy of sorafenib and ICIs. The GSE109211 dataset, namely the phase 3 STORM trial, contained the transcriptome data and therapeutic outcomes of 140 HCC patients receiving sorafenib treatment [25]. Thus it was applied to the sorafenib-related analysis. Regarding ICIs, we addressed the issue from four perspectives, namely tumor mutation burden (TMB), TIDE (Tumor immune dysfunction and exclusion) algorithm, the expressions of immune checkpoints (ICs), and the IMvigor 210 cohort. Among these, TIDE algorithm is pivotal for predicting the response to anti-PD-1/L1 and anti-CTLA4 treatments based on the estimation of T cell dysfunction and tumor immune evasion, which was achieved by its online tool (http://tide.dfci.harvard.edu/login/) [26]. The IMvigor210 dataset was derived from a real clinical cohort and offered a therapeutic response to atezolizumab (a PD-L1 inhibitor) of 348 patients [27].

### Clinical samples and qPCR

After obtaining informed consent from the patients, 20 pairs of HCC and adjacent normal liver tissues were utilized for confirming the differential expression of DLAT. The study protocol was approved by the Ethics Committees of second affiliated hospital of Xi'an Jiaotong University.

Total RNA was extracted using TRIzol Reagent (TakaRa, Japan). RNA concentration was calculated by the A260/A280 ratio with the aid of Nanodrop 2000 spectrophotometer. Reverse transcription reactions were performed via the PrimeScript RT reagent Kit (Takara, Japan). RT-qPCR reaction was marked by SYBR-Green PCR Reagent (Takara, Japan) and tracked on the ABI Prism 7900 sequence detection system. GAPDH was employed as the reference gene. The relative gene expression was calculated according to the 2-ΔΔCT method. The list of primer sequences was shown in Supplementary table 4.

### Immunohistochemistry assay

The Formalin-fixed HCC and the paired adjacent normal tissues were embedded in paraffin and cut into 3 mm sections. The clinical specimens were incubated with rabbit polyclonal antibodies of DLAT (1ug/ml, Abcam, USA) at 4° overnight. Secondary antibodies labeled with horseradish peroxidase (1:400, Abcam, USA) were incubated with the sections at room temperature for 1.5 h. Then, each section was stained with DAB reagent, and finally counterstained with hematoxylin.

### Cell culture and transfection

Two hepatocellular cancer cell lines (HepG2 and Huh-7) that were obtained from Procell Life Science&Technology Company (Wuhan, China) were used for further in vitro experiments. HepG2 and Huh-7 cells were cultured in MEM (Minimum Essential Medium) and DMEM (Dulbecco's Modified Eagle Medium) respectively. Each medium was added by 10% FBS (Fetal bovine serum) and 1% P/S (Penicillin/ Streptomycin) (Procell, Wuhan, China). HanHeng Biotechnology (Shanghai, China) designed and synthesized the short hairpin RNA targeting DLAT (sh-DLAT) and the overexpression plasmids (OE-DLAT). Their specific sequences were shown in Supplementary table 5. The lentiviral system created stable transfected cells (HanHeng Biotechnology, Shanghai, China).

### Colony formation assay

Transfected cells at logarithmic growth phase were seeded into 6-well plates with a density of 1 × 10^3^/ per well. After the incubation of 2 weeks, cell colonies were visible and were fixed by methanol. Giemsa was applied to stain the cell colonies. Finally, colonies were counted under the microscope from five random fields.

### Transwell assays

Transwell assays followed the similar procedures described previously [28]. In migrative assays, the mediums with different concentrations of FBS were added into upper (0.1%) and lower (10%) chambers respectively. Cells were cultured for 24 h, and we used PBS and swab to remove non-migrative cells. Then, the migrated cells were fixed by paraformaldehyde for 20 min and stained by 0.1% crystal violet for 20 min. Cell counting was conducted using a high magnification microscope (100-fold) from five random visual fields. For the invasive assays, the upper chambers were precoated with Matrigel (Corning, NY, USA).

### Statistical analysis

All statistical analyses were performed using the R software (version 4.2.0) and GraphPad Prism (version 8.0). Differences between groups were compared by unpaired T test or Wilcoxon rank sum test. Correlations between CR risk score and the clinicopathological features of HCC were determined via the Kolmogorov–Smirnov test. The in vitro experiments were performed in triplicated. Statistical significance was set at *P* < 0.05.

## Results

### Construction of a novel CR risk signature based on Lasso regression analysis

**Fig. 1:**
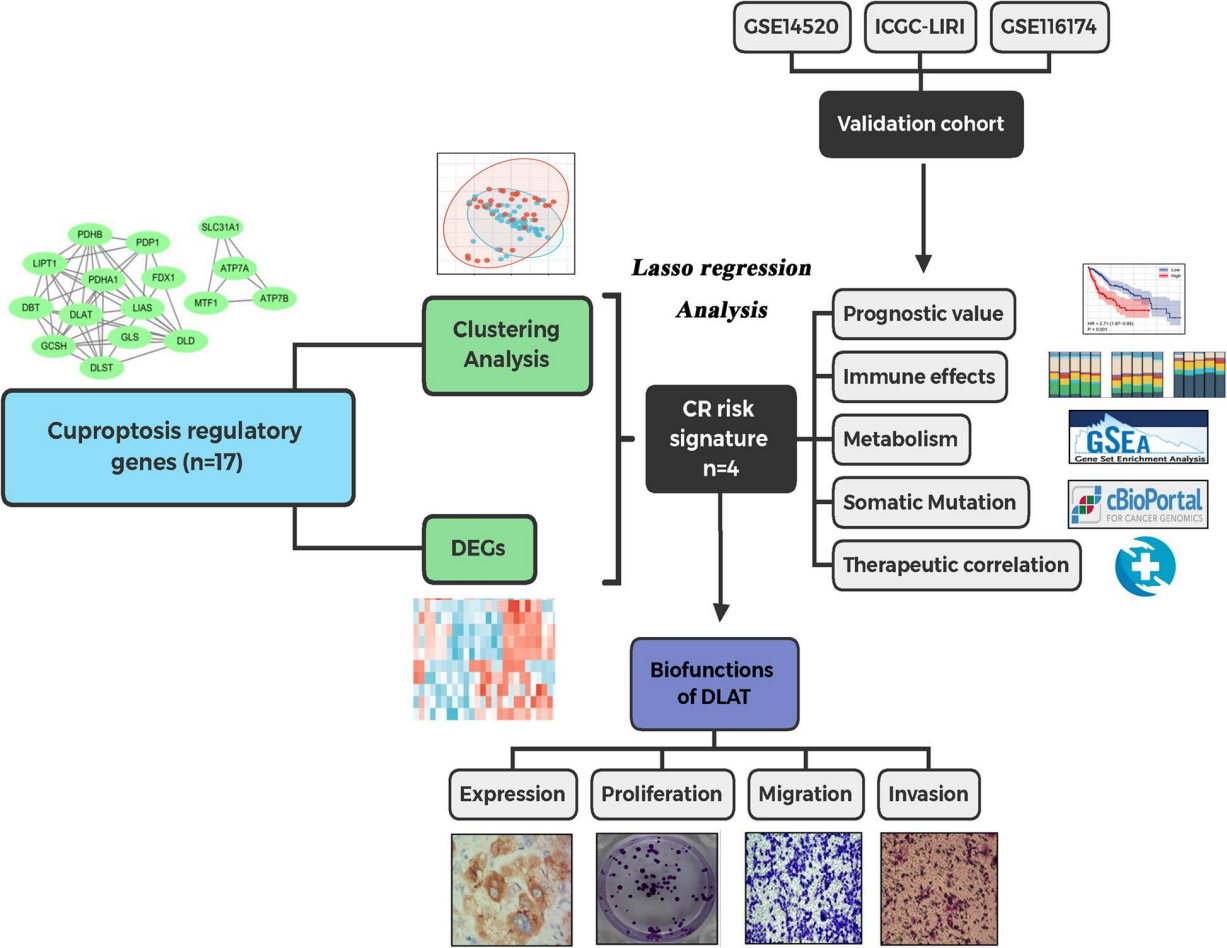
The flow chart of the present study. Diagrams after the item labels refer to the thumbnail images of analytical results or the core methods used. DEGs, differentially expressed genes; CR, cuproptosis-related

The workflow was depicted in Fig. 1. Using CR DEGs, a CR risk signature was constructed through Lasso regression analysis. We comprehensively investigated the roles of the CR risk score in clinical assessments of HCC and selected DLAT, a core cuproptosis regulator, for further investigation.

**Fig. 2:**
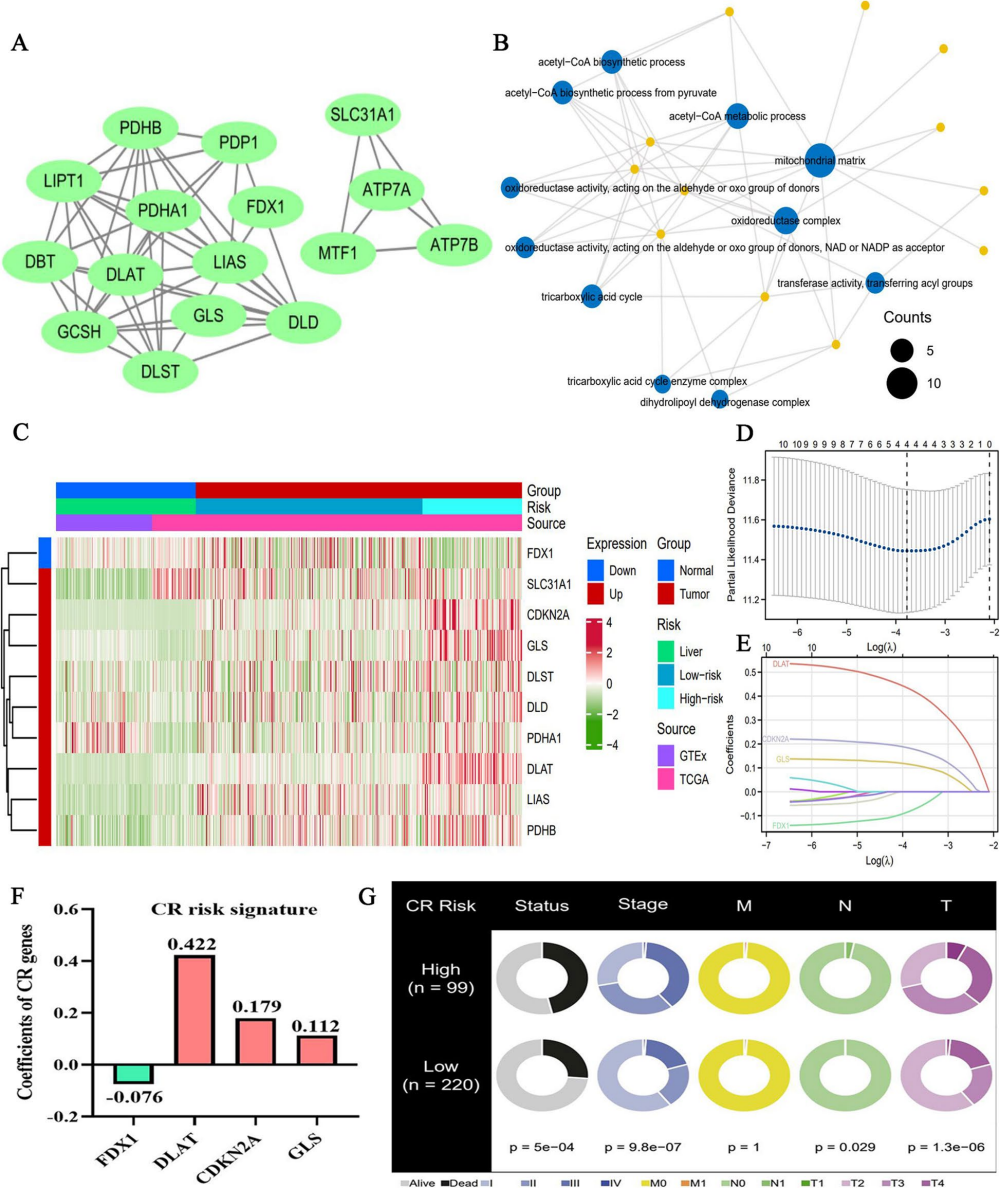
A novel CR risk signature for HCC clinical assessment. **A** The PPI network of 17 CR genes. **B** GO enrichment analysis of 17 CR genes. **C** The expressive heatmap of 17 CR genes. **D** Cross validation process in Lasso regression analysis, which is visualized through the alterations between partial-likelihood deviance (PLD) and Log(λ). **E** Changes in trajectory of variables. With the increase of λ, the coefficient of each variable is decreasing. When the coefficient of some variable attenuates to 0, it is indicative that this variable makes negligible contribution to the model and should be eliminated **F** The coefficients of 4 CR signature genes. **G** The relationships between CR risk levels and clinicopathological features of HCC

According to the detail mechanism of cuproptosis reporting by Tsvetkov P et al. [11], 17 pivotal genes were selected to consist of a CR gene set. The PPI network of these genes was presented in Fig. 2A. Through biological function analysis, we found that these genes were significantly enriched in ‘TCA cycle-related’ processes (Fig. 2B), confirming the tight linkage between cuproptosis and mitochondrial respiration. Among the 17 CR genes, up to 10 genes (58.8%) were differentially expressed in the HCC samples. Their expression heatmap is exhibited in Fig. 2C. Compared with that in the normal samples, FDX1 expression was downregulated and others were upregulated in tumor samples. Through Lasso regression analysis, we constructed a novel CR risk signature. λ value determines which variables could make the model optimal [29]. Cross validation can be used to find the best λ value, which is visualized through the alterations between partial-likelihood deviance (PLD) and Log(λ) [30]. As shown in Fig. 2D, when PLD reached the minimum, we could obtain the best value of Log(λ), which was slightly larger than -4. At this time, the model fitting degree of CR risk signature was also optimal, and its number of variables was 4. Similarly, changes in trajectory of variables also reflect the process of Lasso regression (Fig. 2E). With the increase of λ value, the coefficient of each gene is decreasing. When the coefficient of some gene attenuated to zero, it was indicative that this gene made negligible contribution to the model and should be eliminated. Thus, when Log(λ) took the optimum value (Around -4, Fig. 2DE), there were only four genes whose coefficients did not decay to zero (DLAT, CDKN2A, GLS, and FDX1). Naturally, these genes were applied to construct the CR risk signature, and their coefficients were shown in new Fig. 2F.

According to the novel CR risk signature, the risk score of each HCC patient in TCGA-LIHC cohort was calculated. Through Cutoff Finder online tool [19], we obtained the optimal cut-off value of CR risk score (1.6471), by which 342 HCC patients were divided into high- and low-risk groups (Supplementary Fig. 1). Correlation analysis revealed that survival status, clinical stage, T, and N stages were all closely associated with CR risk levels (Fig. 2G), suggesting that CR risk could reflect the clinical status of HCC patients.

### Two prognostic clusters based on the expressions of CR genes

Subsequently, we stratified HCC patients for prognosis using two cluster patterns based on CR gene expression. Heatmap of the consistency matrix showed that intragroup members were highly homogeneous (Blue module), while intergroup difference was highly obvious (White area), when K-value was 2 (Supplementary Fig. 2A). Similarly, cumulative distribution curve (CDF) revealed that the curve decreased the most gently when K = 2 (Supplementary Fig. 2B). Meanwhile, we observed that the alterations of area under CDF curve were most notable when K-value was 4, while that were second notable when K-value was 2 (Supplementary Fig. 2C). Therefore, above findings pointed that 2 was the appropriate K-value, and HCC patients could be divided into two prognostic clusters in term of the expressions of 17 CR genes. Through survival analysis, we found a significant survival difference between two clusters (Supplementary Fig. 2D). Since CR risk score in cluster 2 was much higher than that in cluster 1 (Supplementary Fig. 2E), high CR risk may be unfavorable to HCC prognosis. Nevertheless, these clustering patterns could only explain 38.2% prognostic variation (Supplementary Fig. 2F).

### Great prognostic value of CR risk signature

**Fig. 3:**
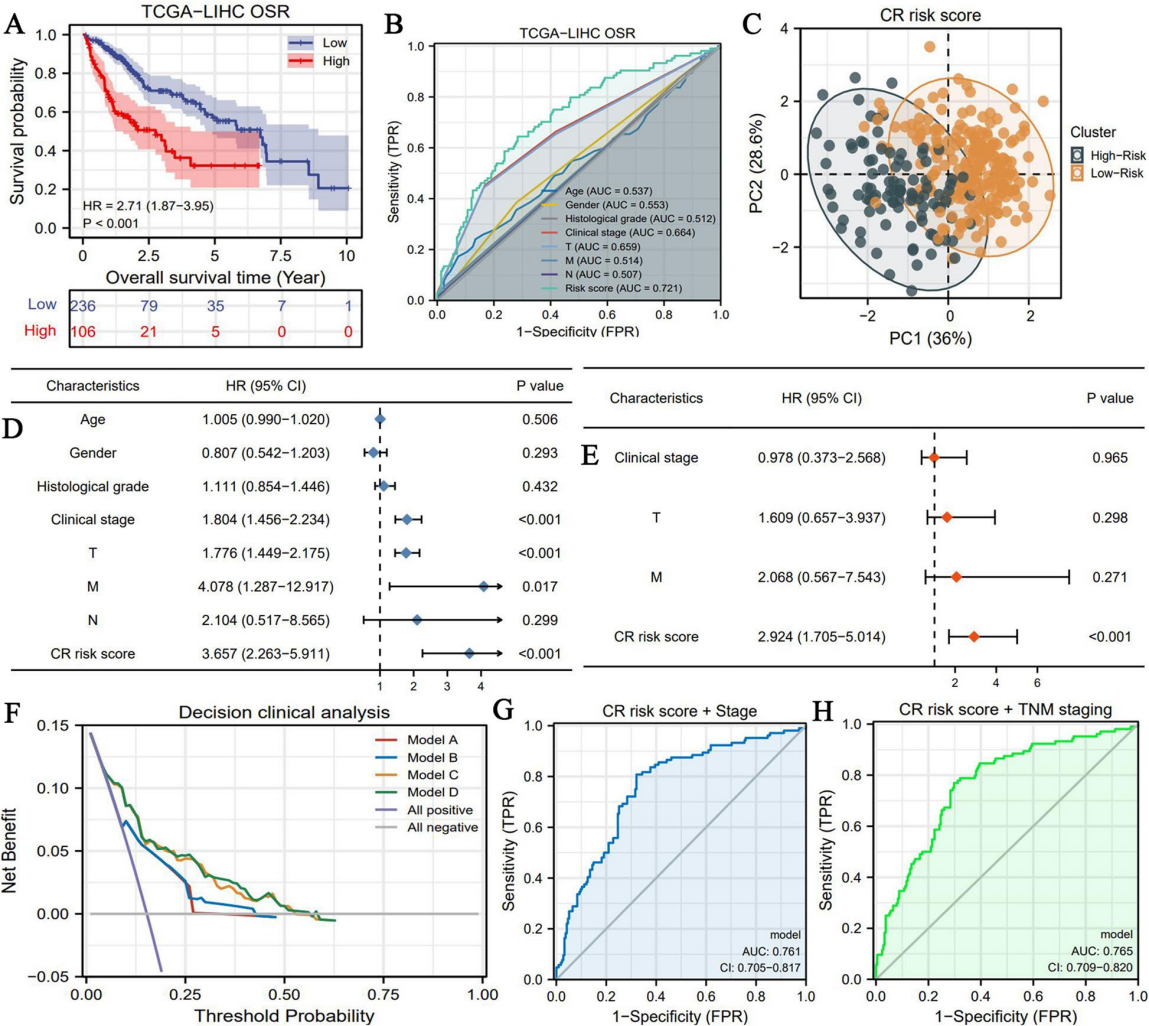
CR risk signature provides critical information for prognostic assessment in HCC. **A** The overall survival difference between high- and low- CR risk groups. **B** The accuracy of CR risk score and other clinical features for predicting OSR. **C** The PCA result of CR risk signature. **D** Cox univariate prognostic analyses. **E** Cox multivariate prognostic analyses. **F** The DCA results. Model A (Red) represents clinical stage model. Model B (Blue) represents TNM-staging model. Model C (Orange) represents the survival model consisting of clinical stage and CR risk score. Model D (Green) represents the survival model consisting of TNM stage and CR risk score. **G** The predictive accuracy of the combination of clinical stage and CR risk score. **H** The predictive accuracy of the combination of TNM-staging and CR risk score. OSR, overall survival rate; HR, hazard ratio; AUC, area under curve; CI, confidence interval

There was an obvious difference in OS between high- and low-CR risk groups. High CR risk conferred a worse prognosis (HR = 2.71, *P* < 0.001) (Fig. 3A). Regarding predictive accuracy, the CR risk score possessed the best performance compared to other clinicopathological features (AUC = 0.721, Fig. 3B). PCA analysis showed that CR risk signature could explain up to 64.6% of prognostic variation; this value was better than that obtained via clustering patterns (Fig. 3C and Sup-Fig. 1F). CR risk score was identified as the only independent prognostic factor of HCC (HR = 2.924, *P* < 0.001) (Fig. 3DE). Notably, through DCA analysis, we found that the combination of TNM-staging and CR risk score or AJCC/Stage and CR risk score could boost the net benefit of single TNM- or Stage-based prognostic models, at a certain risk threshold probability (Fig. 3F). Therefore, the use of the combined prognostic models would greatly reduce decision-errors caused by false-negative or false-positive probabilities. Briefly, CR risk score could increase the clinical decision-making benefit of traditional prognostic models based on clinical stage and TNM system (Fig. 3F). Additionally, CR risk score also enhanced their prediction accuracy (Fig. 3GH). These findings indicated the improvements rendered by the CR risk score to the current prognostic models.

**Fig. 4:**
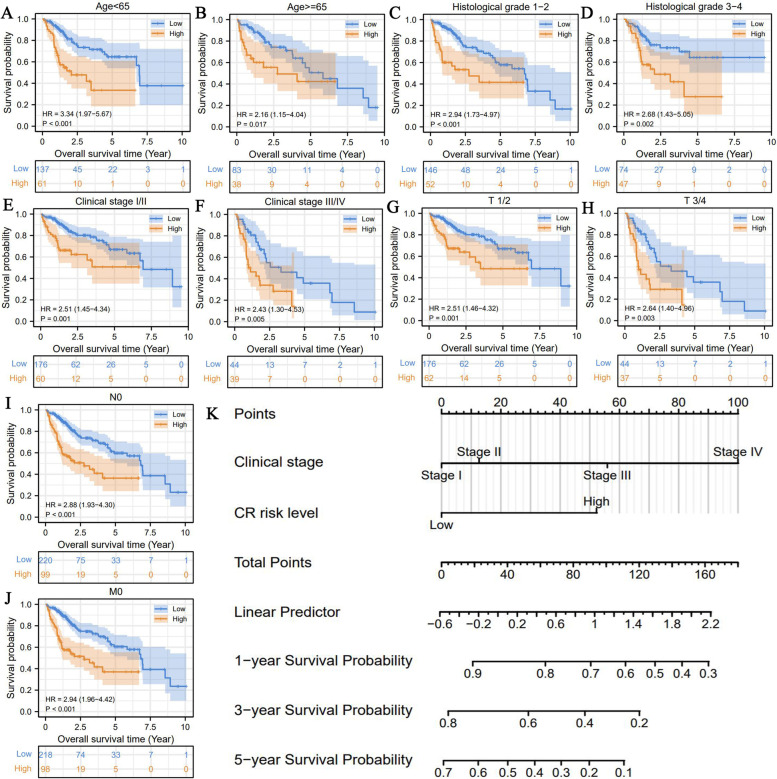
CR risk signature holds wide applicable scope. **A**-**J** The survival differences between high- and low-CR risk groups in each HCC clinical subgroup. **K** The nomogram composing of clinical stage and CR risk level for predicting 1, 3, 5-year OSR of HCC patients

CR risk signature also possessed wide applicability. Clinical subgroup analyses confirmed that CR risk score had potent abilities to distinguish the survival differences of HCC patients in all the subgroups (Fig. 4A-J). Significant prognostic differences were observed between high- and low-CR risk groups irrespective of the stage of disease ( Early stage: T1/2, Stage I–II; Advanced stages: T3/T4, Stage III–IV). Furthermore, we constructed a nomogram comprising of clinical stage and CR risk score to predict the 1-, 3-, and 5-year survival rates of HCC patients in clinical practice (Fig. 4K). For instance, in the same population of clinical stage IV, 1-year OSR of patients with low-CR risk was more than 70%, whereas that of patients with high-CR risk was less than 30%. Additionally, the calibration plots validated the remarkable predictive accuracy of the nomogram (Supplementary Fig. 3).

### Confirmation of the prognostic value of CR risk signature in three validation cohorts

**Fig. 5:**
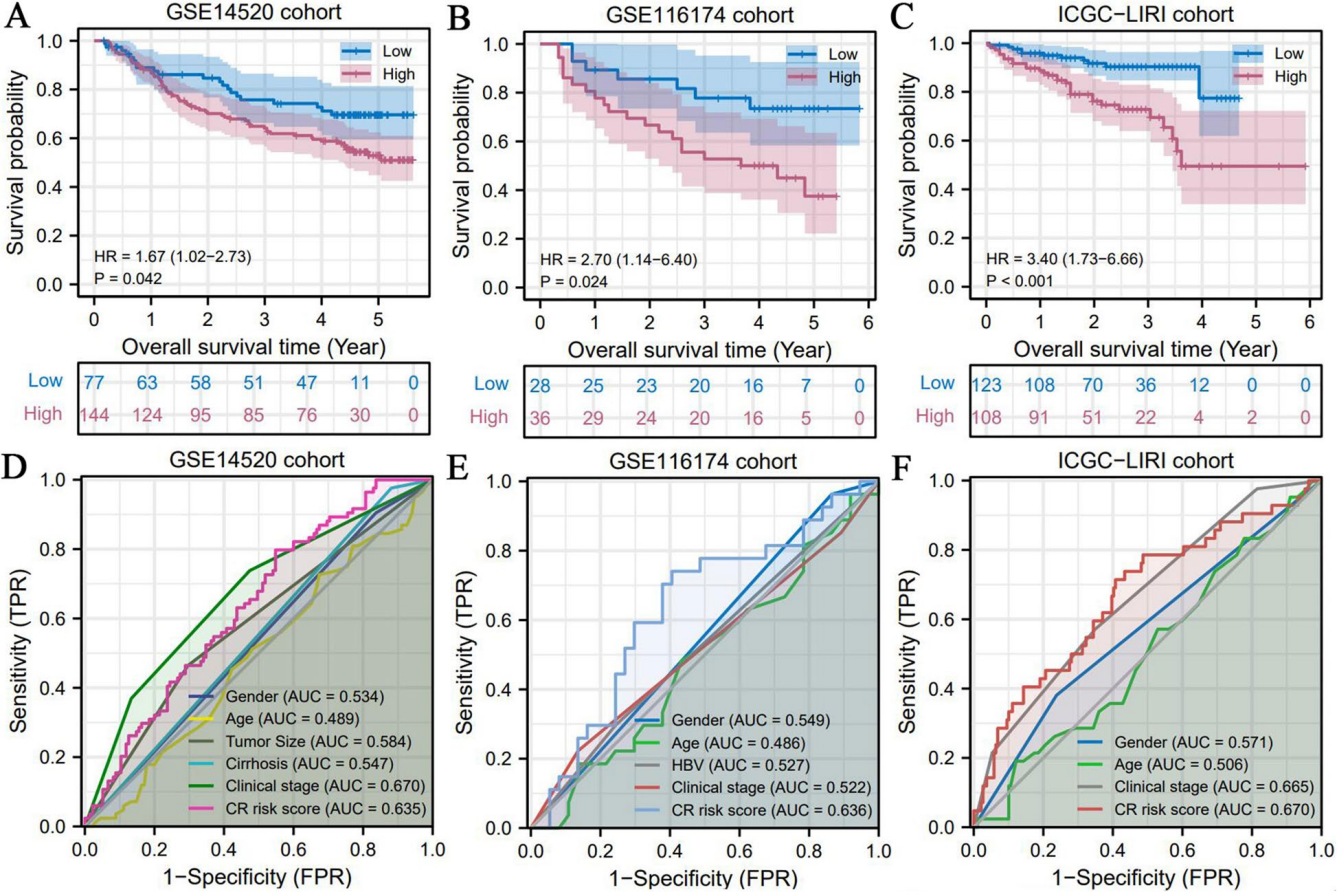
The prognostic value of CR risk signature is applicable to multiple validation cohorts. **A**-**C** The differences in OSR between high- and low- CR risk groups in GSE14520, GSE116174 and ICGC-LIRI cohorts. **D**-**F** The predictive accuracy of CR risk signature in each validation cohort

The aforementioned findings unveiled the prognostic value of CR risk signature in TCGA cohort. Nevertheless, whether it could stratify patients for prognosis in other cohorts remain unelucidated? Our results indicated that there were significant differences in OS between the high- and low-CR risk groups in the GSE14520, GSE116174 and ICGC-LIRI cohorts (Fig. 5A-C). High CR risk led unfavorable survival outcomes. Regarding prediction accuracy, the CR risk score displayed an AUC of 0.635 -0.670 (Fig. 5D-F); this value was moderately weaker than that in TCGC cohort. We found that the CR risk score was superior to other clinical features in predicting OSR of HCC in the GSE116174 and ICGC-LIRI cohorts (Fig. 5E and F). Thus, the CR risk signature was also applicable to other cohorts.

### Associations of adverse anti-tumor immune response with high CR risk

**Fig. 6:**
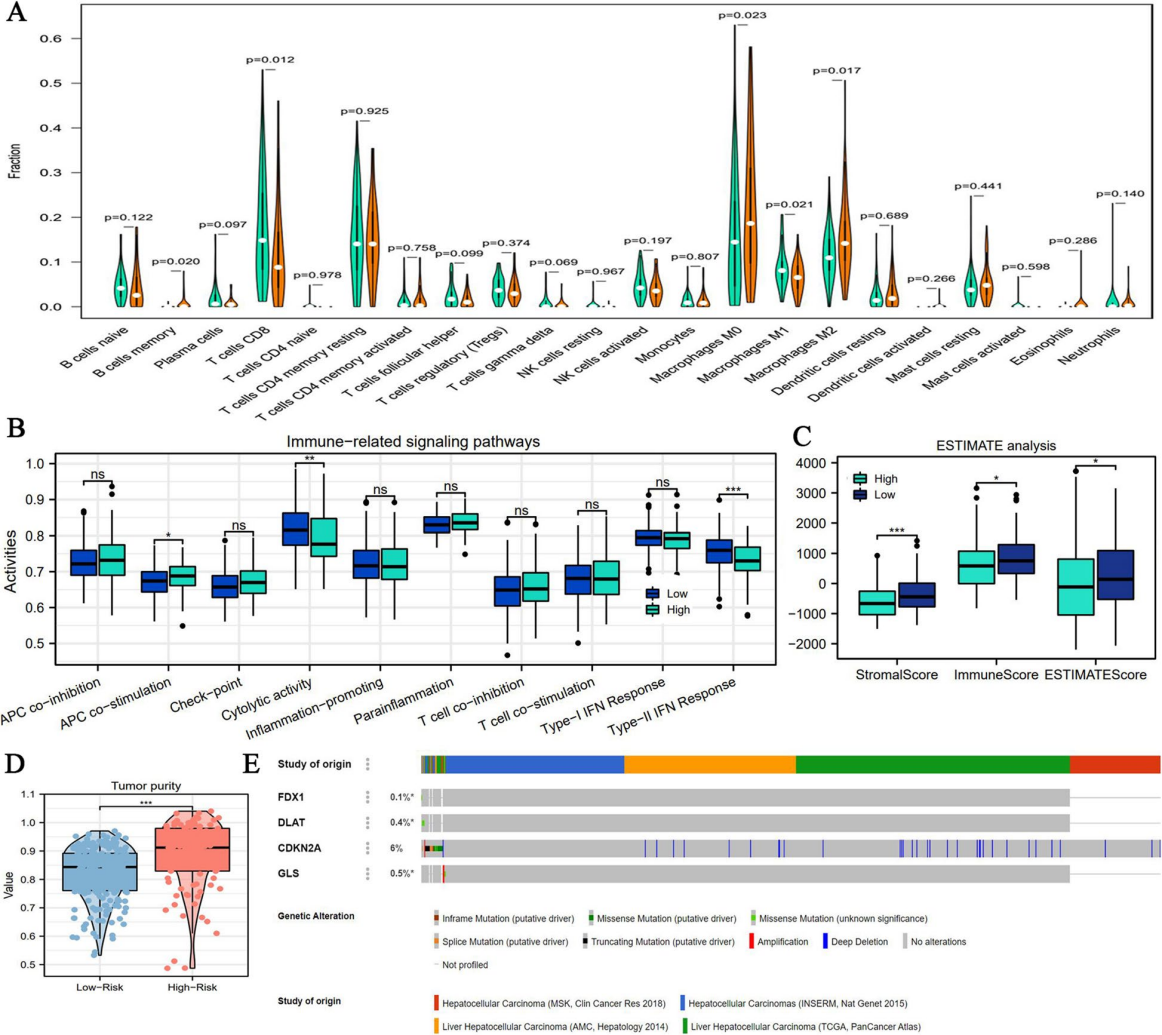
The relationships between CR risk levels and TIM. **A** The differences in infiltration levels of 21 immune cells between high- and low-CR risk levels. **B** The differences in activities of 10 immune-related signaling pathways between different CR risk levels. **C** The differences in immune scores between different CR risk levels based on ESTIMATE method. **D** The difference of tumor purity between different CR risk levels. **E** The somatic mutation information of four CR signature genes based on cBioPortal database. NS, no statistical significance; *P < 0.05, **P < 0.01, ***P < 0.001

Using the CIBERSORT algorithm, the infiltrating abundances of 21 immune cells in each HCC sample were calculated and were presented in Supplementary Fig. 4. High CR risk was accompanied by decreased infiltrating levels of CD8 + T cells and macrophages M1 (Fig. 6A); Conversely, the infiltrating levels of memory B cells, macrophages M0 and macrophages M2 were higher in the high-risk group than that in the low-risk group (Fig. 6A). As shown in Table 2, these alterations in the infiltrating levels were commonly detrimental to the anti-tumor immune process. Similar immune changes were observed in the activity of different pathways. High CR risk was concomitant with the low cytolytic activity and type-II IFN (Interferon) response, but high activity of APC (Antigen presenting cell) co-stimulation (Fig. 6B). ESTIMATE analyses showed that stromal, immune and ESTIMATE scores in low-risk group were significantly higher in the low-risk group than in the high-risk group (Fig. 6C), which revealed that high CR risk was not contributive to the anti-tumor immune process. High CR risk was found to harbor higher tumor purity (Fig. 6D). Therefore, CR risk score could aid in stratifying HCC patients in different anti-tumor immune statuses.

**Fig. 7:**
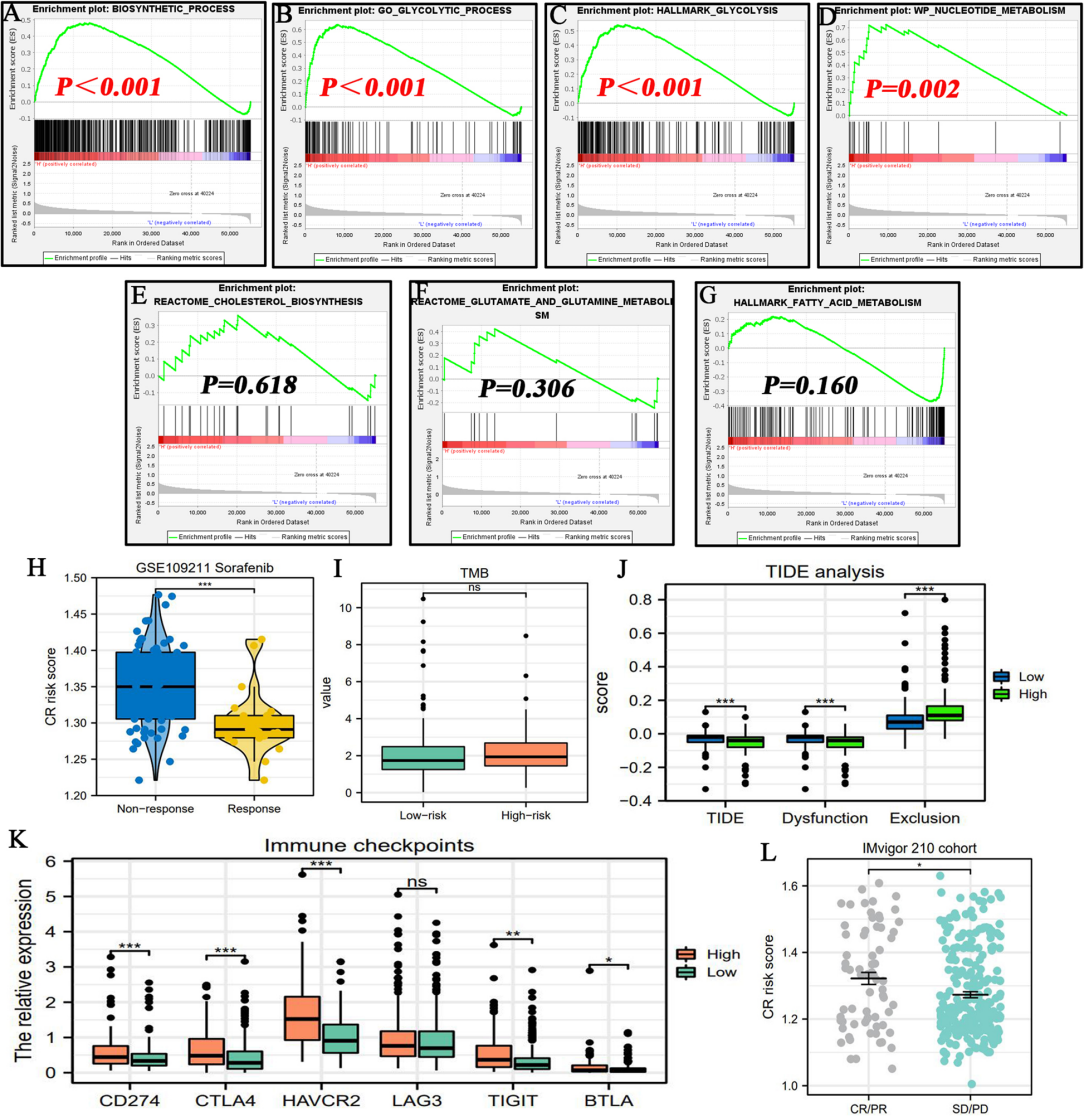
The metabolic and therapeutic correlations of CR risk score. **A**-**G** The associations of CR risk score with the enrichments of multiple metabolic process based on GSEA analysis. **H** The difference in CR risk score between Sorafenib-response and -nonresponse patients based on GSE109211 cohort. **I** The difference in TMB between high- and low-CR risk groups. **J** The results of TIDE analyses. **K** The expressive differences of six ICs between high- and low-CR risk groups. **L** The difference in CR risk score between ICB-response and -nonresponse patients based on IMvigor 210 cohort. ICs, immune checkpoints; *P < 0.05, **P < 0.01, ***P < 0.001

**Table 2:** The effects of high CR risk on tumor immune microenvironment

Immune cell	Variation trend	Roles in tumor immune response	Final effect on antitumor immune
B cells memory	Increased	Memory B cells are critical for the formation of germinal center and plasma cell	Favorable
T cells CD8	Decreased	CD8 + T cells are the most central anti-tumor effectors for their potent cytotoxic effects	Unfavorable
Macrophages M0	Increased	The polarization of macrophage M1/M2 can determine the direction of anti-tumor immunity	Uncertain
Macrophages M1	Decreased	M1 macrophages contribute to eradicating cancerous cells through mediating cytotoxicity and ADCC pathways	Unfavorable
Macrophages M2	Increased	M2 macrophages can suppress the functions of T cells	Unfavorable

*CR* cuproptosis-related, *ADCC* antibody-dependent cell-mediated cytotoxicity

Moreover, the somatic mutation of CDKN2A was most common among all signature genes (6%, Fig. 6E), whereas that of other signature genes was barely visible (< 0.5%, Fig. 6E) in the HCC samples. These results implied that the abnormal expressions of FDX1, DLAT and GLS may result from transcriptional or post-translational regulations.

### Metabolic enrichment of glycolysis indicated by CR risk score

Metabolic reprogramming well represented by glycolysis exerts critical roles in tumor biology. As shown in Table 3, glycolysis, nucleotide, cholesterol, glutamine and fatty acid (FA) metabolisms were closely involved in cancer onset and development. We found that multiple glycolysis-related pathways were significantly enriched in HCC samples with high CR risk (Fig. 7A-C). The same trend in enrichment was found in nucleotide metabolism (Fig. 7D), indicating that high CR risk marked active cell proliferation. However, CR risk levels did not affect the enrichments of cholesterol, glutamine and FA metabolisms (Fig. 7E-G).

**Table 3:** The effects of CR risk levels on multiple metabolic pathways

Metabolic pathway	Enriched phenotype	Study (PMID)	Function in cancer
Glycolysis	High CR risk	32631382	Glycolysis is closely associated with proliferation, immune evasion, invasion, metastasis, angiogenesis, and drug resistance in cancers
Nucleotide Metabolism	High CR risk	34138729	Nucleotide metabolism is necessary for tumor proliferation and mediates oncogenic mutations, such as P53 mutation
Cholesterol Biosynthesis	NS	34117857	Cholesterol is an essential component of cell membranes. Aberrant cholesterol metabolism enhances malignant behaviors of tumor cells
FA metabolism	NS	23791484	Tumor proliferation requires FAs for synthesis of membranes and signaling molecules
Glutamate and Glutamine Metabolism	NS	23999442	Glutamine participates in energy formation, redox homeostasis, macromolecular synthesis, and signaling in cancer cells

*CR* cuproptosis-related, *FA* fatty acid, *NS* not statistical

### Prediction of the efficacy of sorafenib and ICIs by CR risk score

Using transcriptome data and clinical information from the GSE109211 dataset, we explored the correlation between CR risk score and the therapeutic response to sorafenib. The CR risk score was higher in non-response patients than in response patients (Fig. 7H). Hence, high CR risk may be an indicator of sorafenib resistance.

We then investigated the potential associations of CR risk scores with the efficacy of ICIs. First, there was no significant difference in TMB between high- and low-CR risk levels (Fig. 7I). Second, the high-CR risk group presented lower TIDE score than the low risk group (Fig. 7J). Correspondingly, HCC patients with high-CR risk were less susceptible to suffering from the dysfunction of T cells (Fig. 7J). Hence, this finding supported that high CR risk was suggestive of ICIs response. Third, except for LAG3, the high-CR risk was concomitant with higher expressions of other ICs (Fig. 7K). Given that patients with over-expressions of ICs exhibited a good response to ICIs treatment [31], this finding also supported the deduction of TIDE analysis. Finally, we obtained similar analytical results from the IMvigor 210 cohort. Patients with disease remission had higher CR risk scores than those with disease progression (Fig. 7L). This finding supported the aforementioned deduction. Collectively, except for TMB analysis, all our findings demonstrated that high CR risk was related to ICIs response.

### Promotion of the proliferation, migration and invasion of HCC cells by DLAT overexpression

**Fig. 8:**
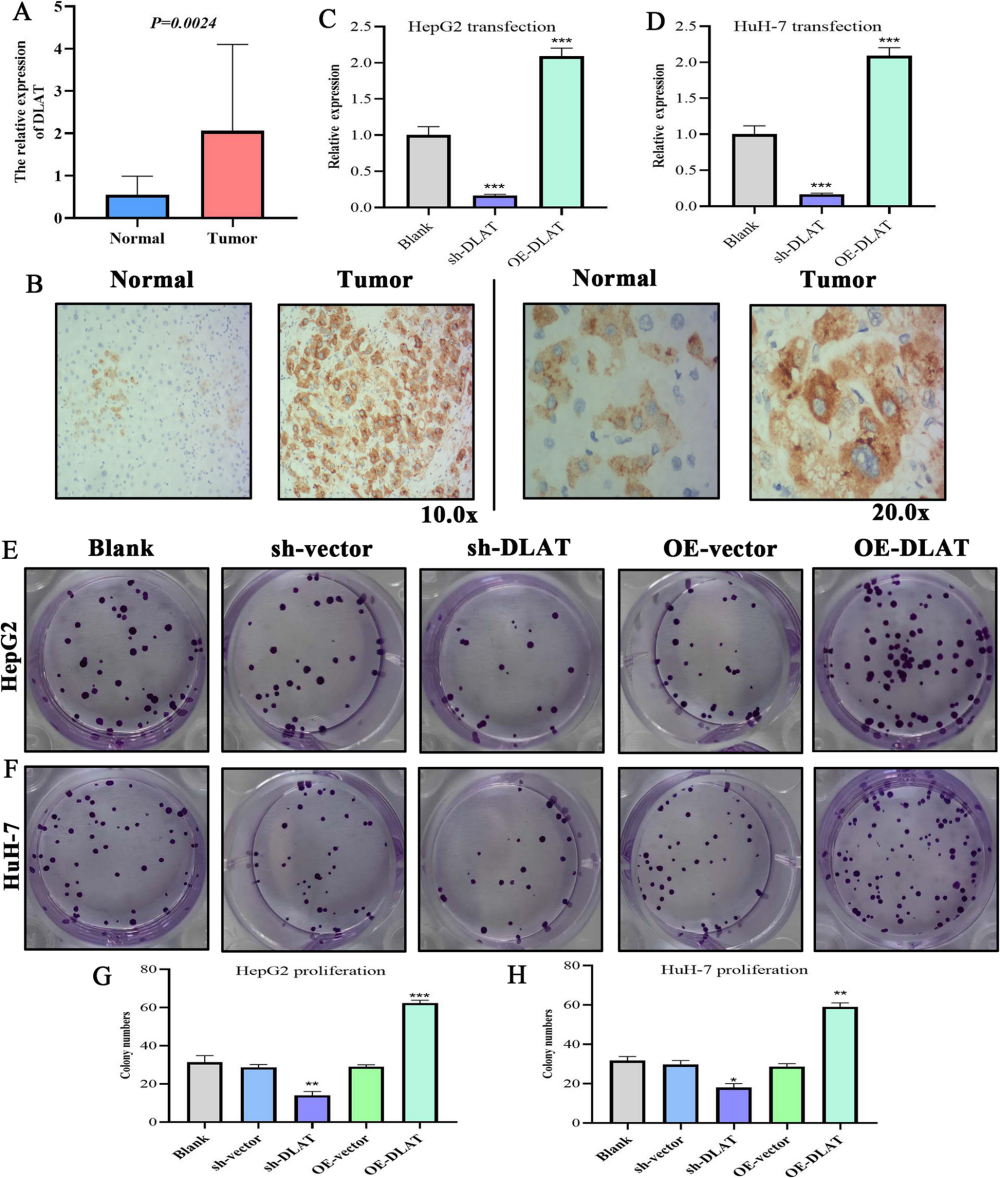
DLAT is overexpressed in HCC tissues and promotes the proliferation of HCC cells. **A** The qPCR detection on 20 pairs of clinical samples. **B** Immunohistochemistry confirmed that the protein expression of DLAT was also upregulated in tumor tissues. **C**-**D** The transfection efficiency of sh-DLAT and OE-DLAT. **E**–**F** The colony formation assays in HepG2 and HuH-7 cells. **G**-**H** The quantitative analysis of colony formation assays. *P < 0.05, **P < 0.01, ***P < 0.001

Although some studies have probed into the roles of CR signature genes in multiple cancers (Table 4), the functions of DLAT remain poorly characterized. We noticed that DLAT exhibited the highest weight of coefficient (0.422) in CR risk signature. Encouraged by these results, we further investigated DLAT. First, we performed qPCR detection on 20 pairs of clinical samples. The mRNA expression of DLAT was significantly upregulated in tumor samples than in the normal ones (Fig. 8A). Moreover, we observed that the protein expression of DLAT was markedly higher in HCC than in normal liver tissues through immunohistochemical analysis (Fig. 8B). sh-DLAT and OE-DLAT effectively altered DLAT expressions in HepG2 and HuH-7 cells, as determined via qPCR tests (Fig. 8C and D).

**Table 4:** The roles of CR signature genes in various cancers

Gene	Study	Cancer type	Function
FDX1	PMID: 32304229	CRC	FDX1 is involved in tumor suppression through mediating TP73 tumor suppressor
	PMID: 36226187	HCC	FDX1 could inhibit the proliferation of HepG2 cells with the presence of copper ions
DLAT	PMID: 26279757	GC	DLAT can promote the proliferation and carbohydrate metabolism of GC cells
	PMID: NA	HCC	Unclear
CDKN2A	PMID: 32594303	CC	CDKN2A inhibits cell proliferation and invasion in CC through AKT/mTOR pathway
	PMID: 28854942	HCC	CDKN2A induces cell cycle arrest in G1 and G2 phases, its mutation is one of the most common molecular anomalies in HCC
GLS	PMID: 24276018	Glioma	GLS silencing synergize with oxidative stress against proliferation of glioma cells
	PMID: 30786811	HCC	GLS promotes cancer progression and metabolism with the mediation of MET

*CRC* colorectal carcinoma, *HCC* hepatocellular carcinoma, *GC* gastric cancer, *CC* cervical cancer

Thereafter, we applied colony formation assays to assess the effects of DLAT on the proliferation of HCC cells. The results showed that overexpression of DLAT notably promoted HCC cell proliferation, whereas it was inhibited by DLAT deletion (Fig. 8E and F). Quantitative analysis results were found to be consistent with the experimental changes (Fig. 8G and H). The colony number was highest in the OE-DLAT group.

**Fig. 9:**
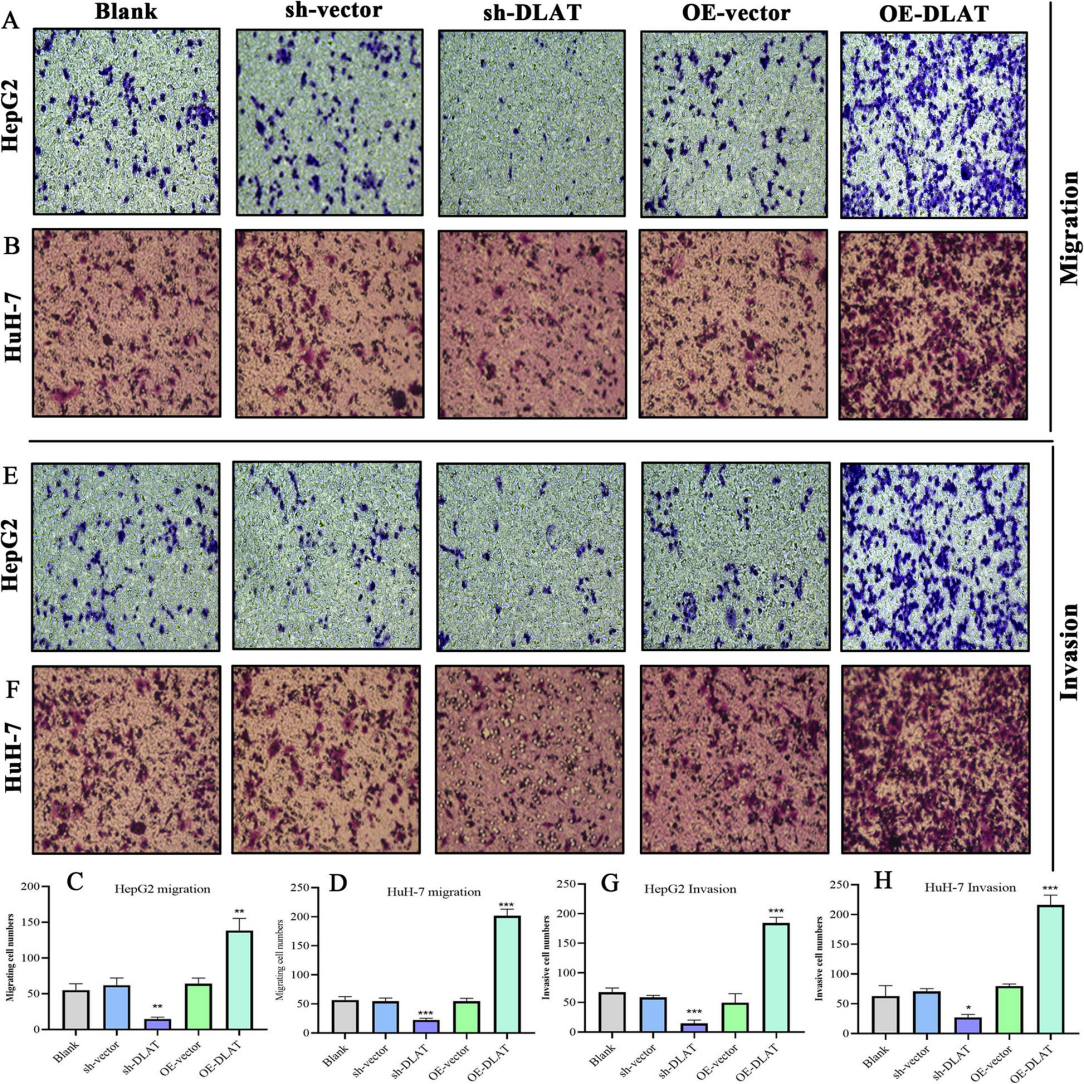
Overexpression of DLAT facilitates the migration and invasion of HCC cells. **A**, **B** The results of Transwell migration assays in HepG2 and HuH-7 cells. **C**, **D** The migrative cells of each experimental group. **E**, **F** The results of Transwell invasion assays in HepG2 and HuH-7 cells. **G**, **H** The invasive cells of each experimental group. *P < 0.05, **P < 0.01, ***P < 0.001

Regarding cell migration, Transwell assays revealed that the overexpression of DLAT had stimulative effects on the migrative abilities of HepG2 and HuH-7 cells (Fig. 9). Conversely, silencing DLAT exerted inhibitory effects (Fig. 9). Similar trend was observed in cell invasion: overexpression of DLAT facilitated the invasion of HCC cells, whereas silencing DLAT suppressed the process (Fig. 9). Altogether, DLAT possessed the promotive effects on the malignant behaviors of HCC cells.

### Comparisons between five existing CR signatures and ours

Since the discovery of cuproptosis, it has attracted substantial attention from mounting oncologists. Regrettably, our study is not the only one to investigate the associations between cuproptosis and liver cancer. Therefore, we discussed the similarities and differences between five existing CR signatures and ours (Table 5) [32–36].

**Table 5:** Comparisons between existing signatures and ours

Study	PMID	Number of CR genes	Model type	Predictive accuracy	Validation cohort	Focus of study	Experimental validation
Zhang G et al	35790864	10	CR LncRNA	0.719	NA	Prognosis ICBs efficiency	NA
Wang X et al	36250008	16	CR genes	0.644	144	Prognosis ICBs efficiency	NA
Wang Y et al	36065073	13	CR genes	0.691	115	Prognosis	NA
Zhang Q et al	36153416	17	CR LncRNA	0.723	175	Prognosis TIM	NA
Zhang Z et al	35898502	FDX1-related	CR genes	0.620	457	Prognosis Sorafenib efficiency	NA
Ours	NA	17	CR genes	0.721	516	Multi-omics	DLAT

*CR* cuproptosis-related, *ICBs* immune checkpoint blockades, *TIM* tumor immune microenvironment, *NA* not available

Through above comparison, some non-negligible preponderances were observed in our study. First, reliable CR gene set. Reasonable establishment of risk signature is heavily reliant on the rigorous gene set. In the present study, we screened 17 pivotal regulators from the cornerstone of cuproptosis research, the study of Tsvetkov P et al*.* [11]. Biological function analysis further confirmed that these genes were tightly involved in the core links of cuproptosis (Fig. 2B). By contrast, Zhang Z et al. have screened out FDX1-related genes (*n* = 200) to construct CR signature by using correlation analysis [36]. However, some critical CR genes were absent in modeling process, such as ATP7A, ATP7B and SLC31A1 which were responsible for copper ion transport [11]. Second, the largest scale of prognostic validation cohort. As shown in Table 5, up to 516 patients from three validation cohorts fully tested the applicability of our CR risk signature in HCC prognostic assessment. Third, more comprehensive bioinformatic analyses. The emphasis of these research was different (Table 5). For instance, Wang G et al. and Wang X et al. focused on the predictive effects of CR signature on the efficacy of ICBs therapy [32, 34]. However, we not only determined whether CR signature could predict the ICBs efficacy, but also investigated its mutational features and functions in anti-cancer immune response and multiple metabolisms, such as glycolysis. Fourth, support of experiment in vitro. Among these research, our study was the only one that verified the biofunctions of CR signature genes in HCC cells. This provided valuable basis for further cuproptosis research. Additionally, our signature exhibited an excellent predictive accuracy, which had preponderance over most studies [32–34, 36]. In this context, although our study was not the only one referring to CR signature, our findings were still instrumental for HCC clinical analysis.

### Potential application of cuproptosis in HCC clinical practice

**Fig. 10:**
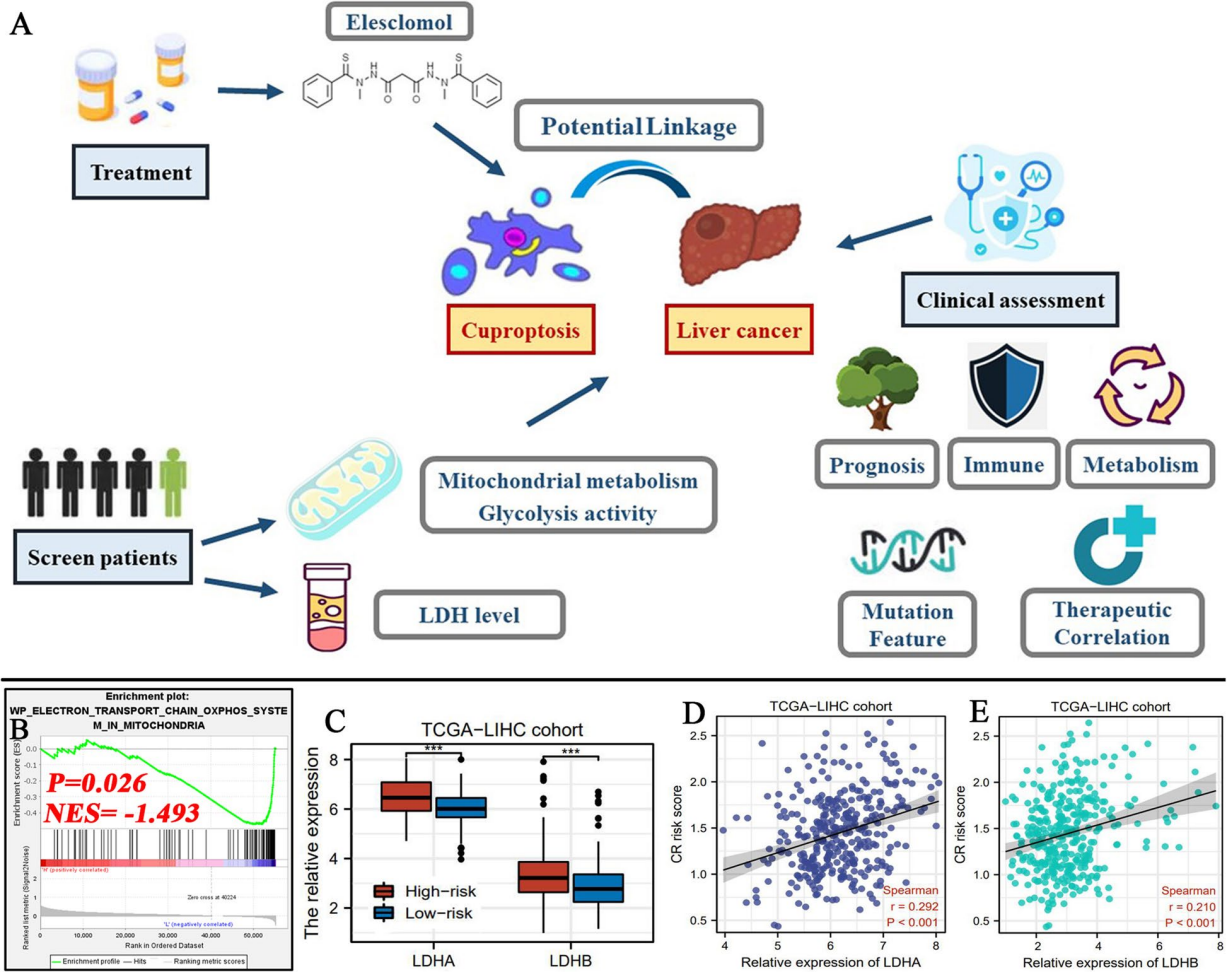
Potential application of cuproptosis in HCC clinical practice. **A** Three possible uses of cuproptosis in HCC treatment and assessment. **B** The GSEA results of elective transport chain OXPHOS system in mitochondria. **C** The differences between in expressions of LDHA and LDHB between two CR risk groups. **D**-**E** Correlations between LDH expressions and CR risk score. NES, normalized enrichment score; OXPHOS, oxidative phosphorylation; LDH, lactate dehydrogenase; ***P < 0.001

As there are no validated biomarkers to detect cuproptosis in humans, how to translate cuproptosis into cancer treatment and assessment should be emphatically probed. In our view, there were three main pieces of evidence witnessing the feasibility of cuproptosis application (Fig. 10A).

First, inducing cuproptosis well represented by Elesclomol (ES) have been proved to be a viable anticancer strategy [37]. ES is a chemotherapeutic adjuvant developed by Synta Pharmaceuticals [38]. Hitherto, multiple clinical trials have confirmed its anti-cancer compacity in human cancers [37]. For instance, a phase II clinical trial revealed that combination of ES and paclitaxel could reduce disease death risk of 41.7% in metastatic melanoma [39]. Meanwhile, introduction of ES doubled the progression-free survival (112 days vs 56 days) [39]. Moreover, another phase I clinical trial targeting refractory solid tumors reported that ES/paclitaxel combination was well tolerated with a toxicity profile similar to single-agent paclitaxel [40]. In view of these facts, cuproptosis enriched the anti-cancer arsenal.

Second, HCC may be sensitive to copper ionophore ES or we have opportunities to screen the population who would benefit from ES treatment. Due to the tight associations of cuproptosis with TCA cycle and cellular respiration, the sensitivity of cancer cells to ES is reliant on the activity of cellular mitochondrial metabolism [37]. A critical piece of evidence derived from the study of Wangpaichitr M et al. [41]. In this study, the activation of mitochondrial respiration was observed in cisplatin-resistant lung cancer cells. Interestingly, cisplatin-resistant cells were more sensitive to ES than parental cells [41]. Furthermore, since mitochondrial respiration and glycolysis commonly hold an antagonistic relationship, active former is indicative of high reliance of cancer cells on anaerobic glycolytic metabolism [42]. Therefore, available evidence have confirmed cancer cells with lower glycolytic activity are also sensitive to ES [43]. Through GSEA analysis, we found that mitochondrial function was significantly enriched in low-CR risk HCC samples (Fig. 10B), whereas glycolysis process was markedly enriched in high-CR risk ones (Fig. 7BC). These findings pointed toward that high-CR risk may act a biomarker of therapeutic resistance to ES in HCC. Notably, some clinical trials have found cancer patients with low LDH levels are sensitive to ES treatment [44]. Phase III SYMMETRY study showed that advanced melanoma patients with low LDH levels had a 1.6-month increase in PFS [44]. Through bioinformatic analysis, we found that the expressions of LDHA and LDHB (The major genes encoding LDH in mammals) were much higher in high-CR risk group than that in low-CR risk one (Fig. 10C). Meanwhile, their expressions increased concordant with CR risk score (Fig. 10DE). These results also supported high-CR risk meant ES resistance.

Third, CR risk score greatly contributed to clinical assessment of HCC patients especially in prognostic analysis (Fig. 10A). Through multi-omics analysis, it was convictive that CR risk levels implied the disease state of HCC patients, which advanced individualized treatments. For example, patients with high-CR risk may suffer from unfavorable prognosis and be concomitant of suppressive anti-tumor immune response, but were probably prone to benefit from sorafenib therapy. Collectively, despite no validated biomarkers to detect cuproptosis in humans, cuproptosis and our CR risk signature still provided some new insights into HCC treatments.

## Discussion

Owing to high malignancy and easy metastases, HCC results in a poor prognosis, with a median survival time of 23 months [45]. Hepatectomy, MTT or ICIs do not fulfill the eager needs of patients for treating liver carcinoma. Recently, the discovery of cuproptosis paints a promising anti-cancer landscape, which may bring a paradigm shift in cancer treatment. Limited available research has reported the roles of cuproptosis regulators in prognosis, immune response and development of cancers, this lack of information prompted us to conduct this investigation.

It is worthy to notice the fact that detective approach of cuproptosis remains obscure, meanwhile no available studies confirmed the existence of cuproptosis in human cancers so far. The most critical issue, whether cuproptosis occurs in HCC needs to be addressed first. We speculated the answer was negative and the following possible reasons resulted in this. First, the accumulation of copper ions couldn’t always trigger cell cuproptosis, but where the copper ions concentrate is the decisive factor [37]. Copper (Cu) is an essential nutrient for a huge number of biological processes including energy metabolism, iron uptake and antioxidant/detoxification processes [46]. Therefore, Cu accumulation has been commonly associated with enhanced proliferation and growth, angiogenesis, and metastasis [46]. Mounting research has determined the upregulation of Cu levels in both serum and tumor tissues in various human cancers such as prostate cancer [47], lung cancer [48] and colorectal cancer [49]. Recently, Tamai Y et al*.* have confirmed that Cu levels was positively correlated with higher BCLC (Barcelona clinic liver cancer) stage in HCC [50]. In light of these findings, Cu levels should elevate in HCC. However, the surge of Cu ions does not directly drive cuproptosis occurrence in HCC. The core reason is the aggregated location of Cu ions. The most critical evidence is the anticancer mechanism of Elesclomol (ES), the only cuproptosis inducer available. Unlike other copper ionophores, ES could selectively promote cellular copper levels in mitochondria, not just in the cytosol [51]. In conclusion, cuproptosis did not occur spontaneously in liver cancer, but active Cu metabolism and high Cu levels in HCC created its precondition. Indeed, two current copper-related anticancer strategies support the above discussion [12]. On one hand, researchers have applied Cu chelators to inhibit Cu-dependent cellular proliferation, termed ‘cuproplasia’, through decreasing the intracellular Cu concentration [52]. On the other hand, Cu ionophores being developed exhibit a promising anticancer direction through stimulating Cu concentration in mitochondria, namely inducing cuproptosis [12].

Accurate prognostic assessment is the most critical component of individualized cancer treatment. Although some mainstream prognostic systems strongly contribute to predicting survival outcomes of HCC patients, these systems are not without their limitations. For example, the Barcelona Clinic Liver Cancer (BCLC) system is insufficient in providing precise distinguishability for prognostically stratifying HCC patients with the intermediate stage [53]. Moreover, AJCC 8th edition staging system fails to discriminate survival differences between patients with IVA and IIIA stages [54]. Giannis D et al. have reported that AJCC 8th edition presented a mediocre predictive ability in a SEER cohort, particularly the advanced TNM stage was not associated with increased risk of death [55]. Thus, improving the existing models is necessary and meaningful. In the present study, the novel CR risk signature could markedly elevate the decision-benefit and predictive accuracy of the AJCC system (Fig. 3F-H), demonstrating that it acted as an essential supplement to the AJCC system. Moreover, CR risk signature was capable of distinguishing the survival difference of III/IV stage cases, which were the inadequacies of AJCC system [54]. Thus, our findings validated the remarkable prognostic value of the CR model.

The alterations in tumor immune microenvironment (TIM) profoundly determine the trend of anti-tumor response. Immune analyses revealed that CR risk score was closely associated with the infiltration level of CD8 + T cells and macrophages. The potent anti-cancer potency of CD8 + T cells has long been known, this immune guarder eradicates tumor cells through perforin and Fas/Fasl pathways [56]. Under different chemokine stimulation, macrophages can differentiate into M1 and M2 subtypes. Macrophages polarization is strongly involved in the cancer immune regulation [57]. Specifically, M1 subtypes can directly target cancer cells, whereas M2 subtypes can drive immune evasion and tolerance by suppressing the functions of CD8 + T cells [58]. Therefore, decreased immune abundance of CD8 + T cells and M1 macrophages (Fig. 6A), and increased that of M2 macrophages resulting from high CR risk all pointed toward unfavorable changes to the anti-cancer immune process. CR risk score may be indicative of anti-tumor response.

Immune checkpoint inhibitors (ICIs) well represented by pembrolizumab (PD-1 inhibitor) have changed the paradigm of cancer treatment. Currently, the NCCN (version 2021) guidelines have listed nivolumab, pembrolizumab and atezolizumab as the first-line option for HCC treatment [59]. Nevertheless, it is inconclusive of identifying a reliable and effective biomarker for predicting the efficacy of ICIs. Here, we found that a high CR risk score may be an indicator of the response to immunotherapy (Fig. 7H-L). Despite the negative result observing in TMB, given that inadequate stimulation for neoantigens formation, high cost of determination and false-negative response population [60, 61], whether TMB is a valid predictor is controversial [62]. In contrast, high expression levels of ICs [63, 64], low TIDE score [26], and analytical results of the IMvigor 210 cohort supported the associations between CR risk score and ICIs efficacy.

Evidence suggests that metabolic reprogramming is a critical hallmark of cancer biology. Particularly, aerobic glycolysis termed the ‘Warburg effect’ widely participates in malignant progression, therapy resistance and immune tolerance of various cancers [65, 66]. Owing to meeting the metabolic requirements of cell proliferation [67, 68], active glycolysis commonly implies cancer development. In this study, we observed that glycolysis was enriched in HCC samples with high CR risk scores (Fig. 7A-D), thereby indicating that glycolysis may be the metabolic driving force of high-risk progression.

Some studies have investigated the functions of CR signature genes in multiple cancers. For instance, FDX1 can promote ATP production and is a risk indicator for LUAD prognosis, but cannot affect the proliferation and apoptosis of LUAD cells [69]. CDKN2A promoter methylation was associated with an elevated HCC risk and indicated HCC progression [70]. GLS as a crucial substrate of MET kinase can promote the metabolism and biogenesis of HCC cells [71]. Nonetheless, only a few studies have reported the roles of DLAT in cancers, which prompted us to conduct further analysis. Through in vitro experimentations, DLAT was established to have pro-oncogenic capacities in HCC, thereby indicating its potentials as an anti-cancer agent. This gene encodes component E2 of the multi-enzyme pyruvate dehydrogenase complex (PDC) and its overexpression leads to cirrhosis and liver failure [72]. Hence, targeting DLAT can also aid in treating other liver diseases.

Nevertheless, there are some limitations that cannot be neglected in this study. First, the CR risk signature requires further validation in a clinical cohort. Second, the specific cancer-promoting mechanism of DLAT in HCC remains elusive. Third, we did not detect the intensity of intracellular cuproptosis at different expression levels of DLAT. Fourth, since cuproptosis research is still in its infancy, lacking the detective means of cuproptosis is a currently unavoidable drawback, which extremely limits the clinical implications of our study. Thus, utilizing cuproptosis for the clinical assessment and treatment of HCC is a long but promising way.

## Conclusions

Cuproptosis has greatly widened the strategies of cancer treatment and is expected to be a new anti-cancer strategy. However, research concerning about this topic is extremely limited. Thus, to this end, we constructed a novel CR risk signature for HCC clinical assessment in the present study. The CR risk score exhibited great prognostic value and provided pivotal supplement to the AJCC prognostic system. Moreover, it was indicative of anti-tumor response and glycolysis metabolic enrichment. Furthermore, it acted as a potential biomarker for predicting the efficacy of sorafenib and ICIs. Finally, owing to the critical functions of DLAT in cuproptosis, we conducted further investigation on its biofunctions in HCC. Through a series of in vitro experiments in vitro, DLAT was validated to possess cancer-promoting abilities. We believe that our findings will provide valuable information for further studies on cuproptosis and HCC.

## Supplementary Information

**Additional file 1: Supplementary figure 1.** The risk plots of CR signature.

**Additional file 2: Supplementary figure 2.** Consensus clustering analyses based on the expression of 17 CR genes. (A) Heatmap of the consistency matrix. When K-value was 2, intragroup members were highly homogeneous (Blue module), while intergroup difference was highly obvious (White area). (B) Cumulative distribution curve (CDF). When K-value was 2, the curve decreased the most gently, indicating 2 was the appropriate K-value. (C) Alterations of area under CDF curve. (D) The difference in overall survival between two cuproptosis clusters. (E) The difference in CR risk score between two cuproptosis clusters. (F) PCA result of cuproptosis clusters. ***P＜0.001.

**Additional file 3: Supplementary figure 3.** The calibration plots.

**Additional file 4: Supplementary figure 4.** The infiltration levels of 21 immune cells in each HCC sample. HCC, hepatocellular carcinoma

**Additional file 5: Supplementary table 1.** The clinical characteristics of TCGA and ICGC cohorts.

**Additional file 6: Supplementary table 2.** The clinical characteristics of GSE14520 and 116174 cohorts.

**Additional file 7: Supplementary table 3.** The detailed description of the gene sets used in GSEA.

**Additional file 8: Supplementary Table 4.** The primer lists.

**Additional file 9: Supplementary Table 5.** The specific sequences of sh-DLAT and OE-DLAT.

## Data Availability

The datasets used and/or analyzed in the current study are available from the corresponding author upon reasonable request. All databases in the present study are open, as follows: TCGA database (https://portal.gdc.cancer.gov/). GEO database (https://www.ncbi.nlm.nih.gov/geo/). ICGC database (http://dcc.icgc.org/). GTEx database (https://xenabrowser.net/datapages/). STRING online tool (https://string-db.org/). DAVID online tool (https://david.ncifcrf.gov/). Cutoff Finder online tool (http://molpath.charite.de/cutoff). cBioPortal database (http://cbioportal.org). MSigDB database (https://www.gsea-msigdb.org/gsea/msigdb/). TIDE scoring system online tool (http://tide.dfci.harvard.edu/login/).

## Abbreviations

BCLC: Barcelona Clinic Liver Cancer
CR: Cuproptosis-related
CDF: Cumulative distribution function
DCA: Decision curve analysis
DEGs: Differentially expressed genes
GSEA: Gene set enrichment analysis
HCC: Hepatocellular carcinoma
ICIs: Immune checkpoint inhibitors
ICs: Immune checkpoints
MTT: Molecular target therapy
OSR: Overall survival rate
PCD: Programmed cell death
PDC: Pyruvate dehydrogenase complex
PPI: Protein–protein interaction
ROC: Receiver operating characteristic curve
TIM: Tumor immune microenvironment
TMB: Tumor mutation burden
TIDE: Tumor immune dysfunction and exclusion
