# Supplementary figures and images for: Cuproptosis regulatory genes greatly contribute to clinical assessments of hepatocellular carcinoma
Source: BMC Cancer. 2023 Jan 7;23:25. doi: 10.1186/s12885-022-10461-2 (PMC9824945; doi:10.1186/s12885-022-10461-2)

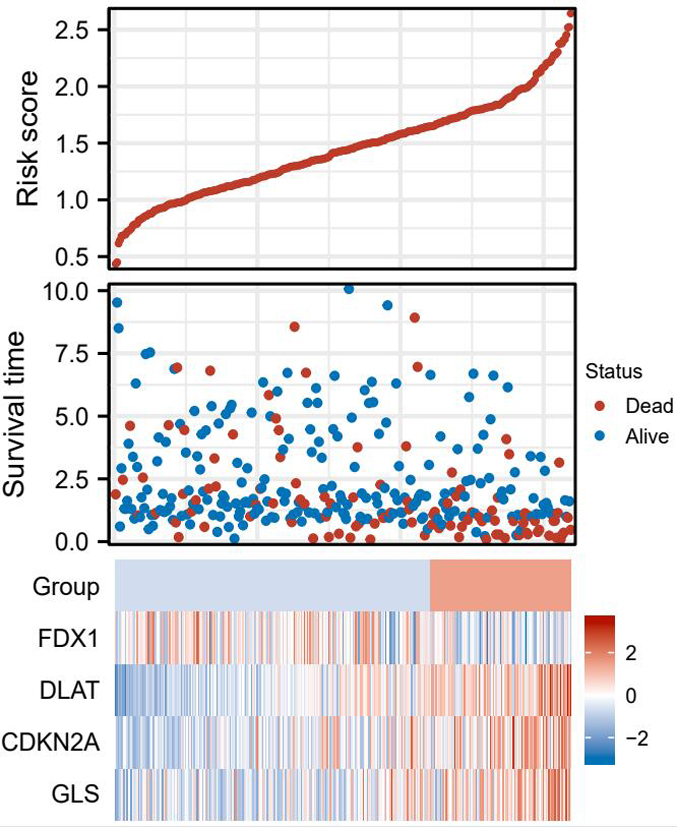

Supplement: Supplementary file 1 — Additional file 1: Supplementary figure 1. The risk plots of CR signature. [file 12885_2022_10461_MOESM1_ESM.jpg]

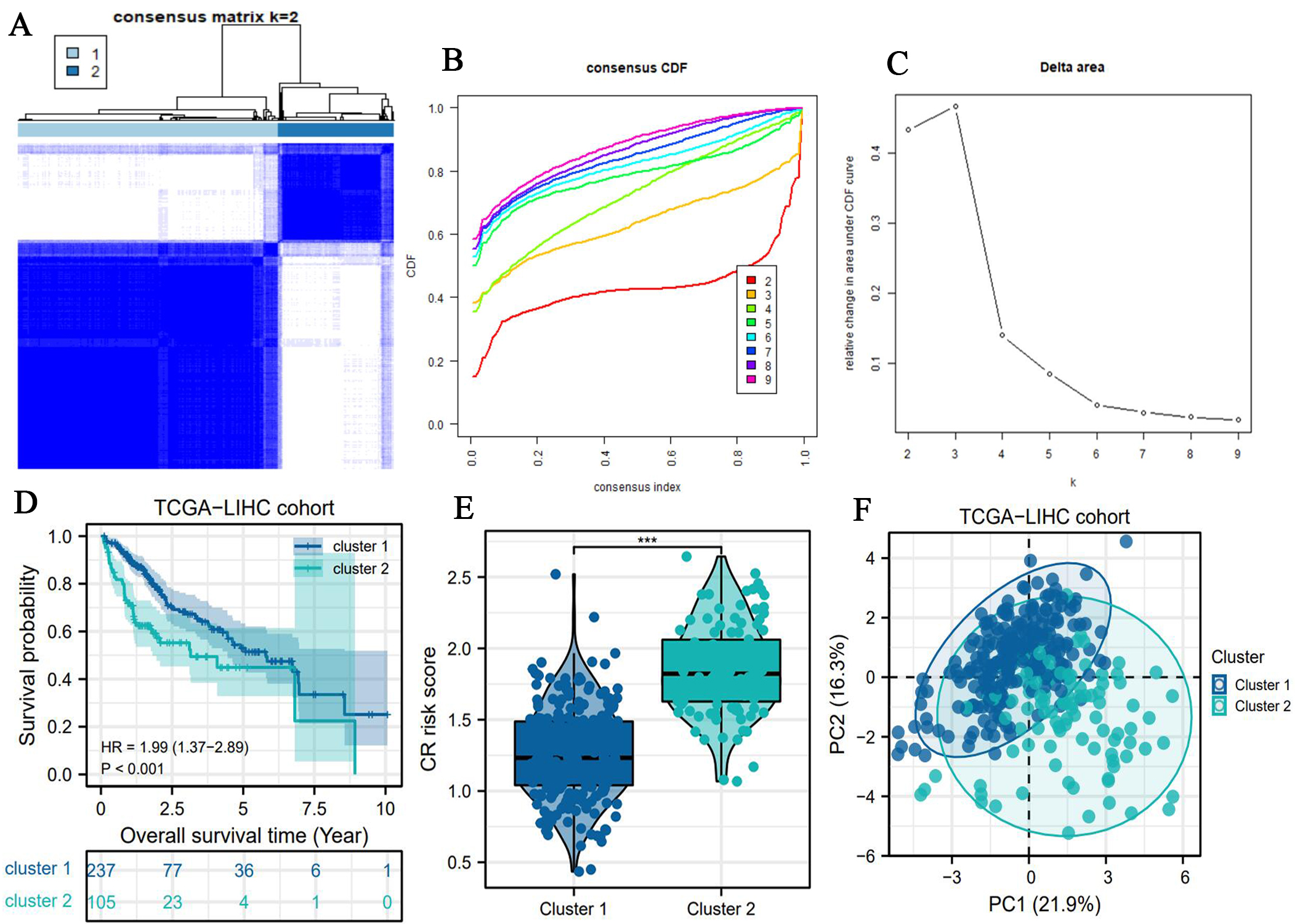

Supplement: Supplementary file 2 — Additional file 2: Supplementary figure 2. Consensus clustering analyses based on the expression of 17 CR genes. (A) Heatmap of the consistency matrix. When K-value was 2, intragroup members were highly homogeneous (Blue module), while intergroup difference was highly obvious (White area). (B) Cumulative distribution curve (CDF). When K-value was 2, the curve decreased the most gently, indicating 2 was the appropriate K-value. (C) Alterations of area under CDF curve. (D) The difference in overall survival between two cuproptosis clusters. (E) The difference in CR risk score between two cuproptosis clusters. (F) PCA result of cuproptosis clusters. ***P＜0.001. [file 12885_2022_10461_MOESM2_ESM.jpg]

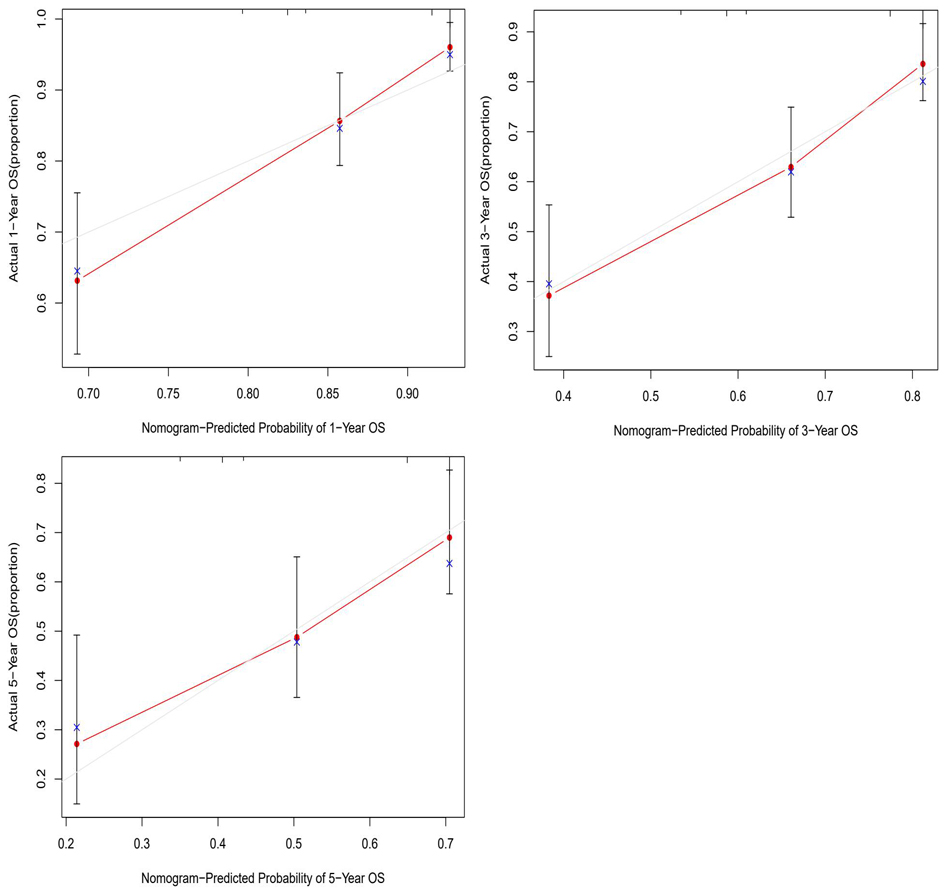

Supplement: Supplementary file 3 — Additional file 3: Supplementary figure 3. The calibration plots. [file 12885_2022_10461_MOESM3_ESM.jpg]

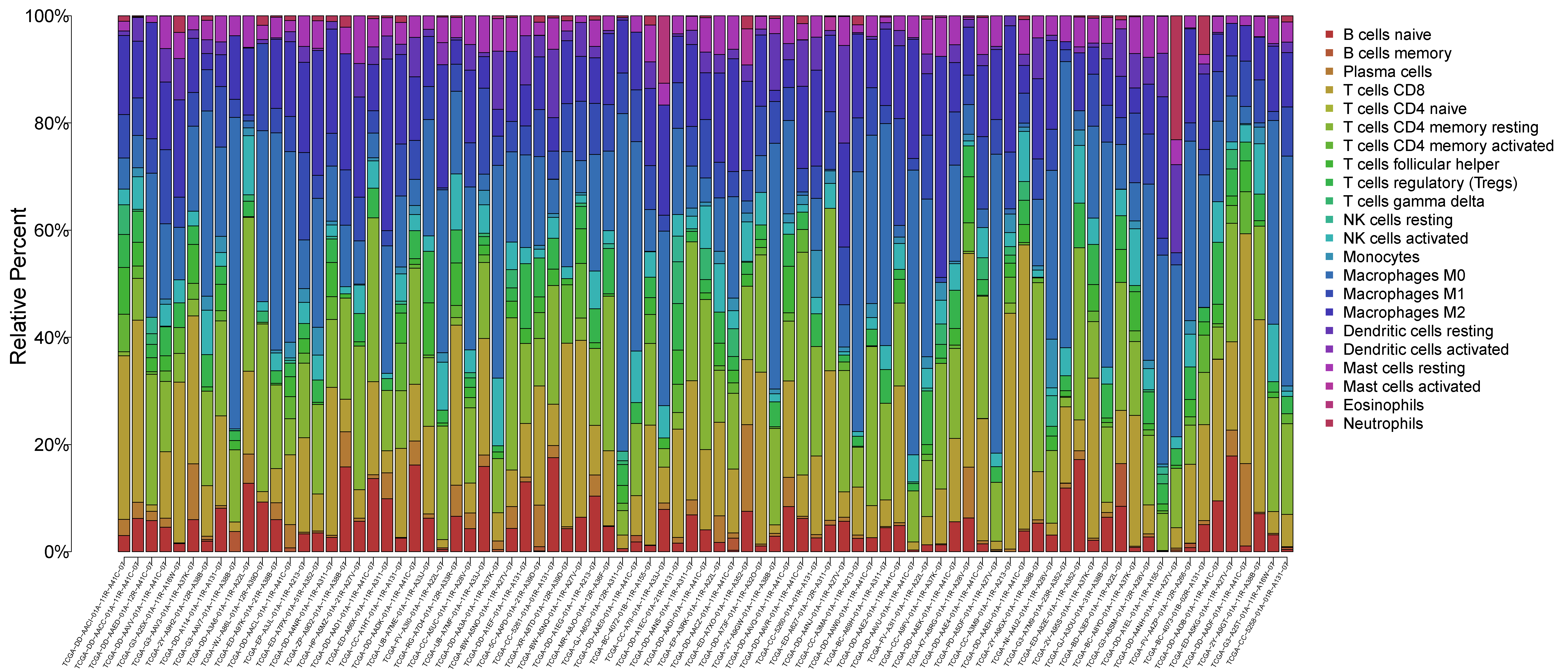

Supplement: Supplementary file 4 — Additional file 4: Supplementary figure 4. The infiltration levels of 21 immune cells in each HCC sample. HCC, hepatocellular carcinoma [file 12885_2022_10461_MOESM4_ESM.jpg]
