# Supplementary material for: Cuproptosis regulatory genes greatly contribute to clinical assessments of hepatocellular carcinoma
Source: BMC Cancer. 2023 Jan 7;23:25. doi: 10.1186/s12885-022-10461-2 (PMC9824945; doi:10.1186/s12885-022-10461-2)
Supplement: Supplementary file 5 — Additional file 5: Supplementary table 1. The clinical characteristics of TCGA and ICGC cohorts. [file 12885_2022_10461_MOESM5_ESM.docx]

Supplementary table 1. The clinical characteristics of TCGA and ICGC cohorts.

| Items | TCGA | ICGC |
| --- | --- | --- |
| Sample size | 377 | 231 |
| Survival status |  |  |
| Dead | 128 | 42 |
| Alive | 249 | 189 |
| Age |  |  |
| ＜60 | 172 | 44 |
| ≥60 | 204 | 187 |
| Unknown | 1 | 0 |
| Histological grade |  | NA |
| G1 | 55 | / |
| G2 | 180 | / |
| G3 | 124 | / |
| G4 | 13 | / |
| Unknown | 5 | / |
| Clinical stage |  |  |
| Stage I | 175 | 36 |
| Stage II | 87 | 105 |
| Stage III | 86 | 71 |
| Stage IV | 5 | 19 |
| Unknown | 24 | 0 |
| T |  | NA |
| T1 | 185 | / |
| T2 | 95 | / |
| T3 | 81 | / |
| T4 | 13 | / |
| Unknown | 3 | / |
| M |  | NA |
| M0 | 272 | / |
| M1 | 4 | / |
| Unknown | 101 | / |
| N |  | NA |
| N0 | 257 | / |
| N1 | 4 | / |
| Unknown | 116 | / |

NA, not available.
