# Supplementary material for: Cuproptosis regulatory genes greatly contribute to clinical assessments of hepatocellular carcinoma
Source: BMC Cancer. 2023 Jan 7;23:25. doi: 10.1186/s12885-022-10461-2 (PMC9824945; doi:10.1186/s12885-022-10461-2)
Supplement: Supplementary file 6 — Additional file 6: Supplementary table 2. The clinical characteristics of GSE14520 and 116174 cohorts. [file 12885_2022_10461_MOESM6_ESM.docx]

Supplementary table 3. The detailed description of the gene sets used in GSEA

| Names | Gene counts | Description |
| --- | --- | --- |
| Biosynthetic processes | 470 | The energy-requiring part of metabolism in which simpler substances are transformed into more complex ones, as in growth and other biosynthetic processes. |
| GO glycolytic process | 106 | Fermentation that includes the anaerobic conversion of glucose to pyruvate via the glycolytic pathway. |
| Hallmark Glycolysis | 200 | Genes encoding proteins involved in glycolysis and gluconeogenesis. |
| WP Nucleotide Metabolism | 19 | Nucleotide metabolism |
| Hallmark Fatty acid Metabolism | 158 | Genes encoding proteins involved in metabolism of fatty acids. |
| Reactome Cholesterol Biosynthesis | 25 | Cholesterol biosynthesis |
| Reactome Glutamate and Glutamine Metabolism | 14 | Glutamate and glutamine metabolism |
| Hallmark Fatty acid metabolism | 158 | Genes encoding proteins involved in metabolism of fatty acids. |
| WP Elective Transport Chain OXPHOS System in Mitochondria | 103 | Electron transport chain: OXPHOS system in mitochondria |

Oxidative phospholytion, OXPHOS.
