# Supplementary material for: Cuproptosis regulatory genes greatly contribute to clinical assessments of hepatocellular carcinoma
Source: BMC Cancer. 2023 Jan 7;23:25. doi: 10.1186/s12885-022-10461-2 (PMC9824945; doi:10.1186/s12885-022-10461-2)
Supplement: Supplementary file 7 — Additional file 7: Supplementary table 3. The detailed description of the gene sets used in GSEA. [file 12885_2022_10461_MOESM7_ESM.docx]

Supplementary table 2. The clinical characteristics of GSE14520 and GSE116174 cohorts.

| Items | GSE14520 | GSE116174 |
| --- | --- | --- |
| Sample size | 221 | 64 |
| Survival status |  |  |
| Dead | 85 | 27 |
| Alive | 136 | 37 |
| Age |  |  |
| ＜60 | 178 | 44 |
| ≥60 | 43 | 20 |
| Clinical stage |  |  |
| Stage I | 93 | 8 |
| Stage II | 77 | 11 |
| Stage III | 49 | 45 |
| Stage IV | 0 | 0 |
| Unknown | 2 | 0 |
| TNM-staging Unknown | / | / |
