# Supplementary material for: Cuproptosis regulatory genes greatly contribute to clinical assessments of hepatocellular carcinoma
Source: BMC Cancer. 2023 Jan 7;23:25. doi: 10.1186/s12885-022-10461-2 (PMC9824945; doi:10.1186/s12885-022-10461-2)
Supplement: Supplementary file 8 — Additional file 8: Supplementary Table 4. The primer lists. [file 12885_2022_10461_MOESM8_ESM.docx]

Supplementary Table 5. The specific sequences of sh-DLAT and OE-DLAT

| Gene | Sequence (5' -> 3') |
| --- | --- |
| sh-DLAT | CCGGGCAGAGGTTGAAACTGATAAACTCGAGTTTATCAGTTTCAACCTCTGCTTTTTG |
| OE-DLAT | DLAT-XbaI-F:  **GCTCTAGA**ATGTGGCGCGTCTGTGCGCGACGGGC |
|  | DLAT-EcoRI-R:  **GGAATTC**TTACAACAACATAGTGATAGGTTTTTC |

OE, over expression.
