# Supplementary material for: Cuproptosis regulatory genes greatly contribute to clinical assessments of hepatocellular carcinoma
Source: BMC Cancer. 2023 Jan 7;23:25. doi: 10.1186/s12885-022-10461-2 (PMC9824945; doi:10.1186/s12885-022-10461-2)
Supplement: Supplementary file 9 — Additional file 9: Supplementary Table 5. The specific sequences of sh-DLAT and OE-DLAT. [file 12885_2022_10461_MOESM9_ESM.docx]

Supplementary Table 4. The primer lists.

| Gene | Primer | Sequence (5' -> 3') |
| --- | --- | --- |
| DLAT | Forward | 5′- GAGATGTCCCTCTAGGAACCC -3′ |
|  | Reverse | 5′- ACAAACACCCTTCCCTTTGGT -3 |
| GAPDH | Forward | 5'‐GTCGCCAGCCGAGCCACATC‐3 |
|  | Reverse | 5'‐CCAGGCGCCCAATACGACCA‐3' |
